# Comparative evaluation of bovine porous bone mineral

**DOI:** 10.4103/0972-124X.70834

**Published:** 2010

**Authors:** M. Parimala, D. S. Mehta

**Affiliations:** *Department of Periodontology and Implantology, Bapuji Dental College and Hospital, Davangere - 577 004, Karnataka, India*

**Keywords:** Bone, grafts, growth factors, platelet rich plasma, periodontal diseases/therapy, periodontal regeneration, re-entry study

## Abstract

**Background:**

The aim of the present clinical study was to compare the efficacy of bovine porous bone mineral (BPBM) with and without platelet-rich plasma (PRP) for the treatment of periodontal intrabony defects.

**Materials and Methods:**

Twenty eight identical bilateral periodontal intrabony defects were selected from 14 chronic periodontitis patients. The subjects were randomly assigned to test group (BPBM+PRP) or the control group (BPBM). The clinical, radiographic, and intrasurgical (re-entry) measurements were made at baseline and at 9 months postoperatively.

**Results:**

Both treatment modalities resulted in significant reduction in probing depth and gain in clinical attachment level as compared to baseline values. The probing depth reduction was 6.20±1.40 mm in BPBM and 6.60±1.43 mm for the BPBM/PRP-treated sites. The gain in clinical attachment level observed was 4.16±1.05 mm for BPBM and 4.70±0.76 mm for the BPBM/PRP group. Radiographically, there was a significant defect fill (3.83±1.01 mm) for the BPBM group and (4.04±1.77 mm) in the BPBM/PRP group. Similar trend was observed between the two groups in relation to intrasurgical parameters.

**Conclusions:**

The combination therapy (BPBM+PRP) showed more favorable clinical outcome in the treatment of intrabony defects than the BPBM alone group, although the mean difference between the two groups was statistically nonsignificant.

## INTRODUCTION

The combination of various regenerative biologic agents and techniques has recently attracted the interest of researchers in the field of reconstructive periodontal surgery. The periodontal regeneration refers to as the formation of new periodontal ligament fibers, alveolar bone, and cementum and requires an orchestrated sequence of biological events for its successful outcome. The different therapeutic modalities that attempt to enhance these biological events such as cell migration, adherence, growth and differentiation, have the potential to increase the success and predictability of periodontal regenerative procedures (Camargo *et al*, 2009).[1]

A variety of bone graft materials are available for use in periodontal regeneration. The autogenous bone grafts have been referred to as the “gold standard” in osseous grafting procedures. However, the limited availability and the complications associated with the donor sites are its main disadvantages. Hence, allogenic materials (FDBA or DFDBA), xenografts and the alloplastic materials are being the mainstay of periodontal regeneration. Xenografts fulfill most of the criteria of an ideal graft material.[2,3] Currently, there are two available sources of xenografts: bovine bone and the natural coral.

The bovine porous bone mineral (BPBM) is a xenograft prepared by protein extraction of bovine bone, and has the ability to enhance bone formation in periodontal intrabony defects.[4–6] A bovine-derived xenograft has recently been introduced into clinical periodontics and proved to be an effective regenerative material. Also, this material has an unlimited supply, requires no additional site for procurement, and it has a proven safety. It is similar to human bone mineral in inner surface area, porosity, crystalline size and calcium to phosphorous ratio.

Recent advances in tissue engineered techniques have provided opportunities for a new regenerative treatment for periodontal defects.[7,8] Platelet-rich plasma (PRP) is an autologous concentration of human platelets in a small volume of plasma. It has been reported that atleast seven fundamental growth factors are secreted actively by platelets to initiate wound healing.[9] However, of all the polypeptide growth factors, the plateletderived growth factor (PDGF) was found to be more favorable for periodontal regeneration. Therefore, the therapeutic modalities that aim at facilitating periodontal tissue cells to behave in a way that is conducive to periodontal regeneration have potential application in patient care.

The aim of the present study was to compare the clinical effectiveness of BPBM with and without platelet-rich plasma (PRP) in the treatment of periodontal intrabony defects.

## MATERIALS AND METHODS

### Patient selection

Fourteen systemically healthy patients (6 males, 8 females) in the age range of 30–55 years (mean age 45±5 years), were selected from the outpatients at the Department of Periodontology, Bapuji Dental College and Hospital, Davangere, Karnataka.

All the patients were informed about the purpose, course, and duration of the study and, signed an informed consent form. The study was conducted in accordance with the Helsinki Declaration of 1975, as revised in 2000 and 2008, and the study design was approved by our Institution’s ethical committee.

### Study design

The study was designed as a randomized and split-mouth clinical trial, comparing the periodontal treatment outcome of BPBM/ PRP (Experimental Site-A) and BPBM alone (Experimental Site-B) in the treatment of periodontal intrabony defects.

### Selection criteria

The selection criteria included: (1) patients having at least two identical bilateral intrabony defects, (2) the probing pockets depth of ≥ 6 mm following the initial therapy with radiographic evidence of vertical/angular bone loss in the affected sites, (3) systemically healthy patients, (4) patients who had not received any periodontal therapy for the past 6 months, (5) patients who had not used antibiotics within the previous 6 months prior to treatment.

### Presurgical therapy and clinical measurements

All the selected patients underwent Phase 1 therapy. Baseline clinical parameters like plaque index,[10] and the gingival index[11] were recorded. Alginate impressions were made and study casts were prepared for each patient. A customized acrylic occlusal stent with a groove (guide plane) was fabricated for each patient to fit it over the selected sites. Using the groove as a guide, the probing depth (using the gingival margin as a reference) was measured by using William’s graduated periodontal probe, whereas the clinical attachment level and gingival recession were measured using the most apical end of the stent as a reference point with the help of silver points.[12] All the customized acrylic stents were preserved on the prepared study casts throughout the study period to minimize distortion. Intraoral peri-apical (I.O.P.A.) radiographs of each of the selected sites were taken, using the long cone paralleling technique.

### Platelet rich plasma preparation

Platelet rich plasma (PRP) was prepared by using a commercially available system according to the manufacturer’s guidelines. Briefly, one hour before the surgery 10 ml of blood was drawn from each patient by venipuncture of the anticubital vein in the forearm and collected in vacutainer that contained anticoagulant (10% trisodium citrate solution). After centrifugation, the concentrated PRP was then activated with 10% CaCl_2_ and thrombin solution. Within 10 seconds, PRP gel was ready to be mixed with bone graft material.

### Surgical and intrasurgical procedures measurements

The operative site was anesthetized with 2% xylocaine with adrenaline [Livnox, Warren, Indico Remidies Ltd, Mumbai, India.] (1:80,000). After achieving adequate anesthesia, crevicular incisions were given on the facial and lingual/ palatal sides. Using B.P. knife with blade No.12, a full thickness mucoperiosteal flap was reflected using the periosteal elevator, taking care that optimum interdental papillary tissue was preserved or retained. After reflection of the flap, the osseous defects were thoroughly debrided and the roots were planed using the Gracey curettes and 4R-4L.

**Figure 1:**
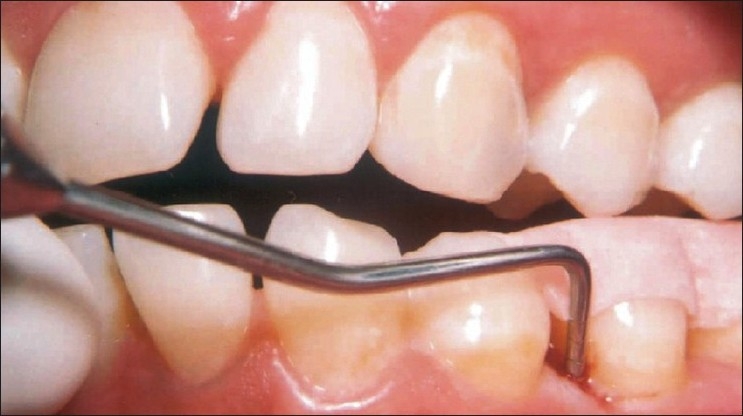
Pre-treatment view of mandibular left second premolar (tooth #35) with 7 mm probing depth on the mesial aspect

**Figure 2:**
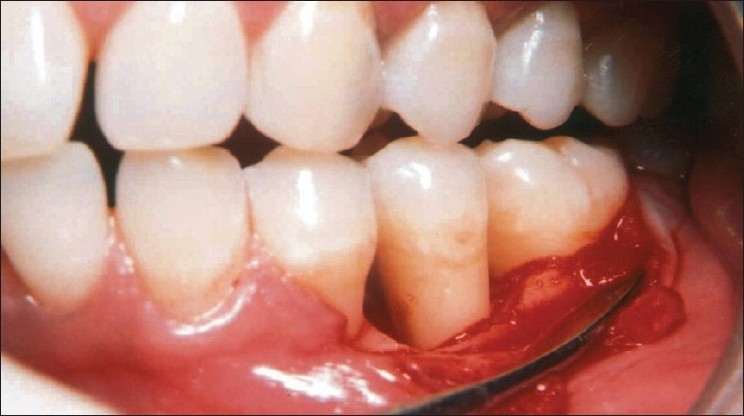
Reflection and debridement of the area revealed a 3 wall defect on the mesial aspect of tooth #35

**Figure 3:**
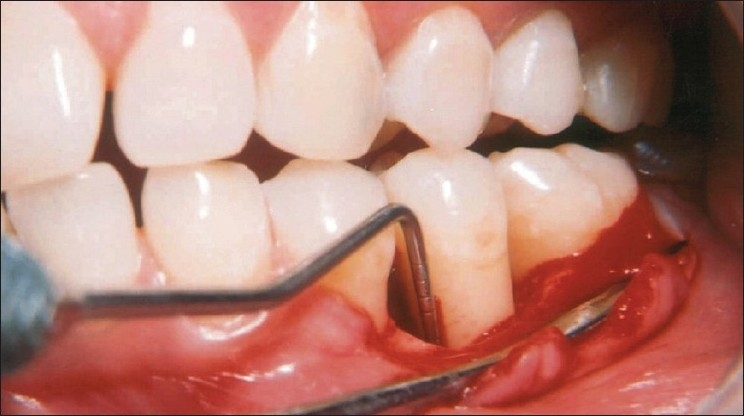
Measuring the defect after debridement revealed osseous defect of 7 mm

**Figure 4:**
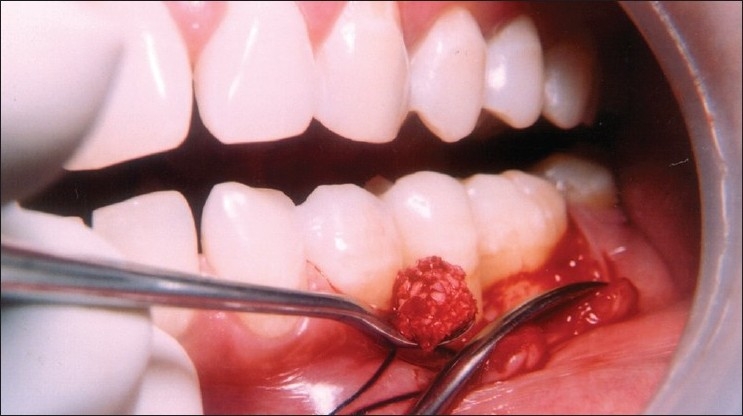
Carrying the composite graft of Bio-Oss/PRP to the defect site

**Figure 5:**
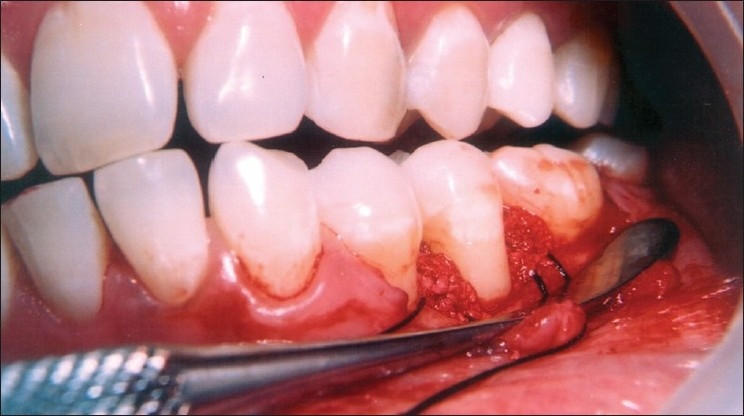
Placement of PRP mixed graft into the defect

**Figure 6:**
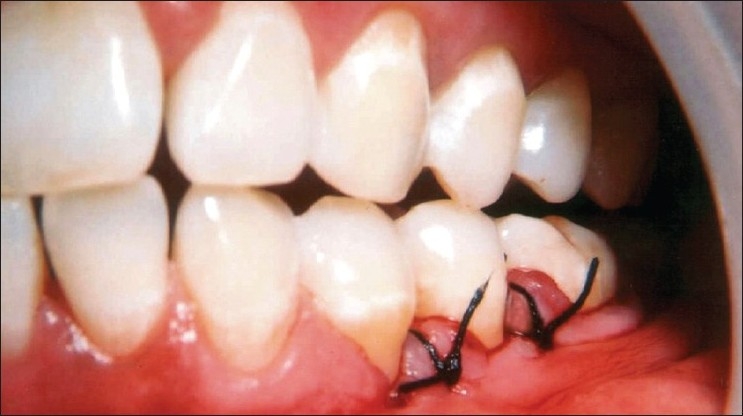
The flaps are sutured to achieve complete wound closure

Columbia curettes (Hu-Friedy). (Hu-Friedy Mfd. Co., Inc, Illinois, Chicago.) The surgical site was then thoroughly irrigated with normal saline and the intrasurgical measurements of the osseous defect were made [Figures 1–3]. The flaps were partially presutured before the placement of the graft material. For experimental site-A, Bio-Oss (BioOss, Geistlich, Wolhusen, Switzerland.)/PRP was delivered into the osseous defect [Figures 4–6] with light incremental pressure using the scoop of a Cumine scaler (Hu-Friedy). However, for Exp. Site-B, the Bio-Oss was mixed with saline and the resultant cohesive mass was placed into the osseus defect. The material was loosely packed from the base of the defect towards coronally. After the defect was filled in both the groups, the presutured mucoperiosteal flaps were repositioned and secured in place using the black braided (4-0) silk sutures (Ethicon, Johnson and Johnson, Aurangabad, India.) The surgical area was protected and covered with noneugenol periodontal dressing. (Coe Pack, GC America Inc, Alsip, Illinois, USA.) All patients were prescribed systemic antibiotic, Doxycycline HCl 200 mg for first day followed by 100 mg/day for 6 days, and an analgesic, Ibuprofen (400 mg) after every 8 hours was prescribed for 3 days. Chlorhexidine gluconate (Orasep, Warren, Elan Pharma, Mumbai, India.) (0.2%) rinse was advised twice daily for first 4 weeks after the surgery.

### Post-operative care

One week following surgery, the periodontal dressing and sutures were removed and the area was irrigated thoroughly with normal saline. Oral hygiene instructions were reenforced. Patient was recalled after 1 week, 1 month, 3 months, 6 months, and 9 months postsurgery and at each visit, oral hygiene status was assessed and scaling was done if necessary.

Nine months postoperatively, all clinical, radiological and intrasurgical (re-entry) measurements were repeated.

### Surgical re-entry procedures

**Figure 7:**
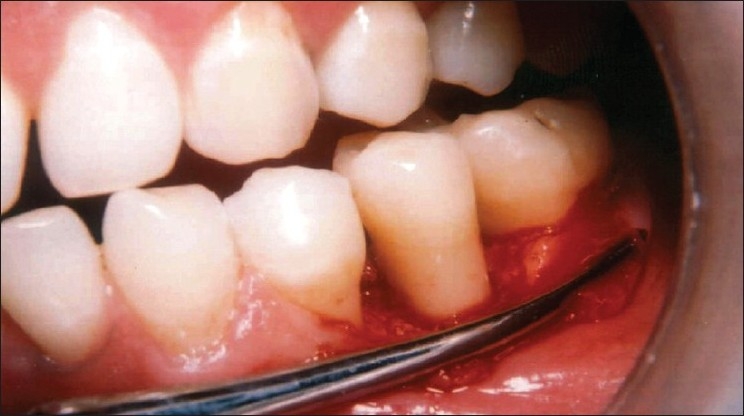
Nine month surgical re-entry reveals hard bone fill in the area of the defect

**Figure 8:**
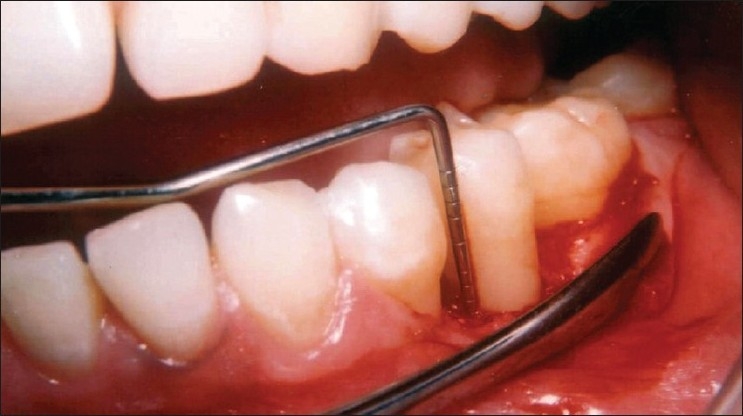
Measuring the defect at nine-month surgical re-entry reveals complete elimination of the defect

After achieving adequate anesthesia, a full thickness mucoperiosteal flap was reflected and the loose soft tissue if present, was removed. All the hard tissue measurements taken at the first surgical procedure were repeated during re-entry surgery using the customized occlusal acrylic stent as described earlier [Figures 7 and 8].

### Radiographic assessment

The conventional I.O.P.A. radiographs were scanned with Hewlett Packard Scan Jet 6300. Computer-assisted densitometric image analysis (CADIA)[13] of the radiographs was done with the help of an image analysis software.

### Statistical analysis

Measurements of both hard and soft tissue dimensions were obtained at the baseline and at 9 month recall (re-entry). The results were averaged (mean±standard error) for each Parimala and Mehta: Bio-Oss with and without PRP in periodontal regeneration parameter. For intragroup comparison, students paired ‘t’ tests were used. However, for comparison between the two groups, two sample rank test (Mann–Whitney ‘U’ test), a nonparametric test was performed.

## RESULTS

All the fourteen patients returned for the re-entry evaluation at the end of 9-month follow-up period. The post-operative healing was uneventful in all the cases. No complications such as allergic reactions, abscesses or infections were observed throughout the study period.

The changes in probing depth (PD) are reported in Table 1. At 9-month post-operative evaluation, there was significant reduction (*P*<0.001) in the mean probing depth in both the therapeutic modalities, when baseline data were compared. However, no significant difference was observed between the two treatment groups.

**Table 1:** Clinical parameters at baseline and 9 months (mm±SD)

Clinical Parameters	BPBM				BPBM / PRP				A vs B
	Base-line (mm)	9 months (mm)	Difference (mm)	*P* Value	Base-line (mm)	9 months (mm)	Difference (mm)	*P* Value	
Probing depth	8.70±1.57	2.50±0.53	6.20±1.40	<0.001*	9.00±1.25	2.40±0.52	6.60±1.43	<0.001*	*P*=0.43 NS
Clinical attachment level	16.12±1.27	11.96±1.59	4.16±1.05	<0.001*	17.15±1.58	12.45±1.25	4.70±0.76	<0.001*	*P*=0.33 NS
Gingival recession	7.47±1.04	8.04±1.13	(-) 0.57±0.76	<0.05*	8.03±1.18	8.89±1.13	(-) 0.86±0.39	<0.01*	*P*=0.47 NS

* Statistically significant; NS Not statistically significant

The clinical attachment level (CAL) improved significantly at 9 month in both the treatment groups (*P*<0.001) when baseline values were compared [Table 1]. Although the BPBM/PRP group showed greater improvements in CAL gain, the difference between the two treatment groups was not significant.

The radiographic assessment at 9-month follow-up period showed statistically significant defect fill and defect resolution in both the treatment groups (*P*<0.001) when baseline values were compared. The alveolar crest height was increased by 1.07±1.47 mm for BPBM and 0.66±1.25 mm for BPBM/PRP- treated group. However, there was no significant difference between the two treatment groups in respect of any of the above parameters [Table 2].

**Table 2:** Radiographic evaluation (Mean±SD)

Clinical Parameters	Bio-Oss				Bio-Oss / PRP				A vs B
	Base-line (mm)	9 months (mm)	Difference (mm)	*P* Value	Base-line (mm)	9 months (mm)	Difference (mm)	*P* Value	
Defect fill	7.59±1.27	3.76±1.24	3.83±1.10	<0.001*	8.48±1.44	4.44±1.57	4.04±1.77	<0.001*	*P*=0.57 NS
Defect resolution	4.07±0.98	1.31±0.43	2.80±0.98	<0.001*	4.63±1.02	1.51±0.43	3.12±1.06	<0.01*	*P*=0.74 NS
Change in alveolar crest height	3.52±1.27	2.45±1.22	1.07±1.47	<0.05*	3.85±1.12	3.19±1.53	0.66±1.25	<0.01*	*P*=0.79 NS

* Statistically significant; NS Not statistically significant

**Figure 9:**
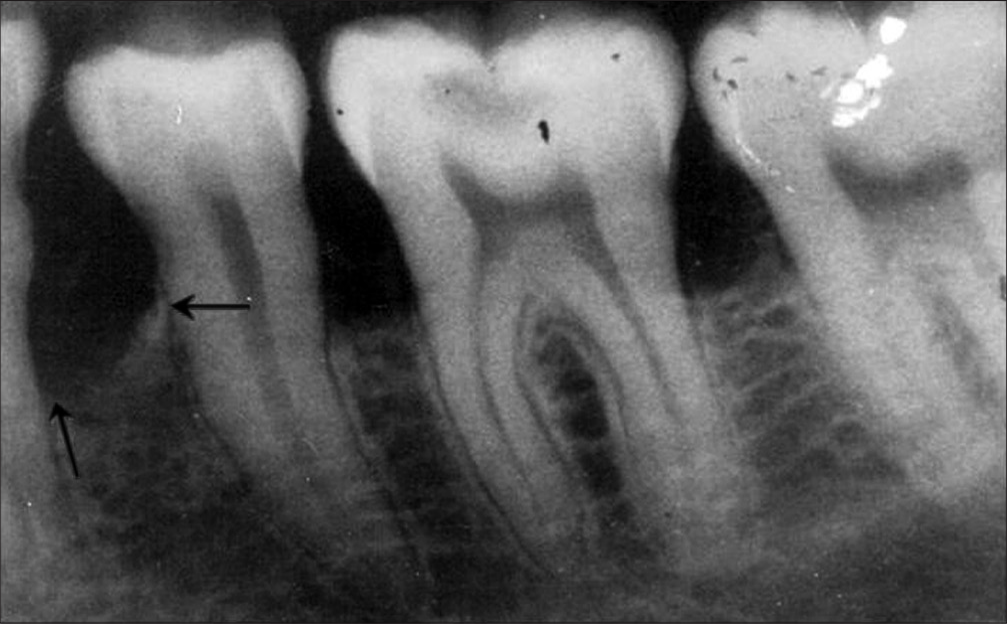
Pre-treatment periapical radiograph of mandibular left second premolar (tooth # 35) showing an inrabony defect on the mesial aspect

**Figure 10:**
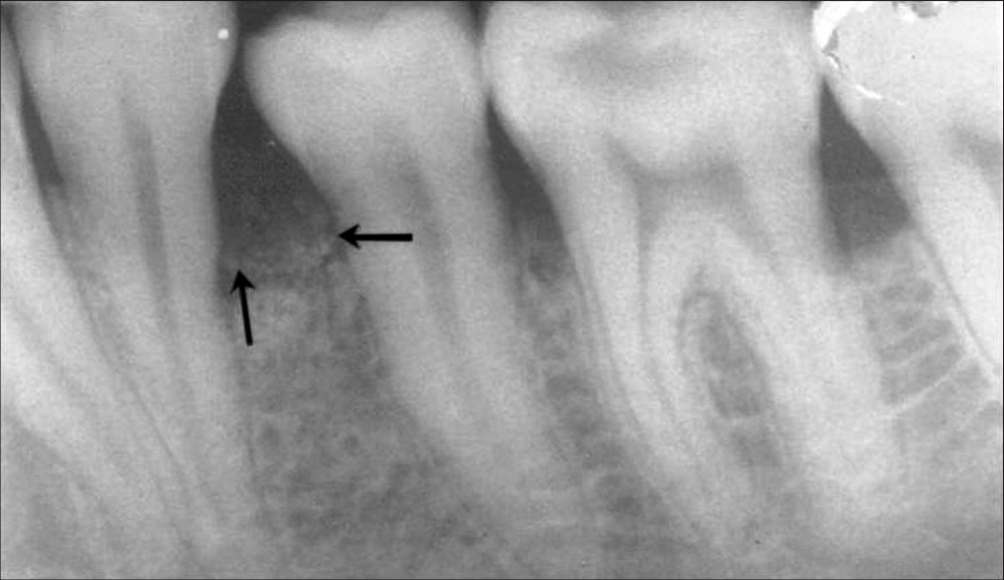
Nine month post treatment periapical radiograph depicts defect fill on the mesial aspect of tooth # 35

On intra surgical evaluation at the 9 month surgical re-entry [Figures 9 and 10], there was significant improvement in defect fill, defect resolution, and gain in alveolar crest height in both the treatment groups. However, the difference between the two treatment groups in respect of above parameters was statistically not significant [Table 3].

**Table 3:** Surgical re-entry (Mean±SD)

Clinical parameters	Bio-Oss				Bio-Oss / PRP				A vs B
	Base-line (mm)	9 months (mm)	Difference (mm)	*P* Value	Base-line (mm)	9 months (mm)	Difference (mm)	*P* Value	
Defect fill	8.80±1.93	3.50±0.77	5.30±1.77	<0.001*	10.10±2.23	4.10±2.23	6.30±1.83	<0.001*	*P*=0.52 NS
Defect resolution	5.70±1.25	1.00±0.94	4.70±1.42	<0.001*	6.80±1.32	1.20±1.32	5.80±1.69	<0.001*	*P*=0.71 NS
Change in alveolar crest height	3.20±1.14	2.50±1.08	0.70±0.48	*P*<0.01	3.40±1.26	3.00±1.49	0.40±0.52	*P*<0.05	*P*=0.34 NS

* Statistically significant; NS Not statistically significant

## DISCUSSION

Predictable regeneration of periodontal osseous defects is a significant challenge in periodontal therapy. Recent studies suggest that when PRP is used in conjunction with various bone derivatives/substitutes, it enhances new bone formation both qualitatively and quantitatively.[1,2] It was also observed that the Bio-Oss/PRP combination showed a greater potential in promoting and improving the clinical signs of periodontal regeneration in infrabony defects in humans.[14] The present clinical study was undertaken to study and compare the effects of BPBM with or without the PRP in the treatment of periodontal intrabony defects.

Three wall infrabony defects were selected because bone regeneration is believed to be improved with an increasing number of bony walls facing the root surface. Three wall osseous defects allow better containment, stability, and increased blood supply to the graft and thought to allow formation of the “space for osteogenesis”.[15] The depth and width of osseous defects also affect the bone regeneration wherein the deep, narrow osseous defects always show better results than the shallow and wide defects. Although it is hard to control all the variables in any clinical study, randomization as done in the present study, may help to control these variables to a greater extent.

Pocket depth resolution is not only a desirable outcome of periodontal regeneration, it may also be the most important parameter commonly used for decision making in patient care for the clinician. It directly relates to the ability of the patient and the clinician to maintain plaque of a non damaging level and also impacts one’s ability to instrument a treated area during the maintenance appointments. In the present study, both the treatment modalities resulted in a significant reduction in probing depth as compared to baseline, similar to the observations made by Camargo *et al*.[16] However, no significant difference in mean probing depth reduction was observed between the two treatment groups. Camargo *et al*,[17] reported significantly better results with BPBM/PRP treated sites as compared to BPBM alone.

The near perfect positive correlation between the gain in the clinical attachment level and gain in alveolar bone height has led to the use of clinical attachment level as an important clinical outcome variable in regenerative studies. The mean gain in clinical attachment level was 4.16±1.05 mm for BPBM and 4.70±0.76 mm for the BPBM/PRP treatment group, which was significant both clinically and statistically. These findings were similar to those reported by Camargo *et al*.[17] and Hanna *et al*.[18] However, on comparison between the two treatment groups, no significant difference was observed in relative gain of clinical attachment level. This seems in agreement with the results of other clinical study.[19]

The radiographic assessment was done by utilizing the computer-assisted image analysis (CADIA) of these radiographs as it gives accurate reading on the digital processing unit.[13] The linear distances from the CEJ to the base of the defect and from alveolar crest to the base of the defect were measured to evaluate the defect fill, defect resolution, and change in the alveolar crest height. Defect fill with new bone formation is a desirable outcome of any periodontal regenerative procedure. At 9 months, the mean radiographic defect fill was 3.83±1.10 mm for BPBM and 4.04±1.77 mm for the BPBM/PRP-treated sites. Similarly, the defect resolution for BPBM and BPBM/ PRP was 2.80±0.98 mm and 3.12±1.06 mm, respectively. The improved results in BPBM/PRP-treated sites can be attributed to the possible role played by growth factors present in the PRP, leading to new bone formation both quantitatively and qualitatively.[17]

The surgical re-entry has been considered as the gold standard for the accurate method of evaluating the hard tissue changes after regenerative therapies.[1,15–17] The main advantage of this technique is that it provides the most definitive information regarding hard tissue response to the therapy. In the present study, there was significant defect fill, defect resolution, and gain in alveolar crest height at 9 month surgical re-entry procedure in both the treatment groups and the results were in agreement to those of Camargo *et al*,[15] Camargo *et al*,.[17] and Lekovic *et al*.[19]

The precise mechanism of action of PRP on periodontal regeneration is not well understood. However, it is suggested that PRP contains high concentration of several growth factors such as PDGF and TGF-β, which may strongly modulate the regeneration process.[14] Invitro studies showed that PRP stimulated the proliferation of periodontal ligament and osteoblastic cells, whereas epithelial cell proliferation was inhibited.[20] Furthermore, because of its fibrinogen content, PRP reacts with thrombin and induces fibrin clot formation that in turn is capable of upregulating collagen synthesis in the extracellular matrix and promotes a favorable scaffold for cellular migration and adhesion. Its “sticky consistency” may also improve the handling properties when combined with bone graft. Also, because of the high fibrin content, it may act as hemostatic agent, which enhances the stability of blood clot and the graft material.[21–23] PRP adheres to the root surfaces and impedes the apical migration of epithelial and connective tissue cells from the flap.

BPBM when used with PRP acts as a scaffold for bone growth, serves to bind PDGF, and localize it to the wound site.[24] Hence, the results in the present study demonstrated that BPBM/ PRP combination is more effective in improving periodontal condition and achieving the periodontal regeneration. These results were in agreement with the earlier studies. However, it appears that the benefit added by combining PRP with BPBM is not statistically significant. Future studies are recommended with larger sample size and longer follow-up.
